# Supplementary material for: An assessment of organisational culture in Australian hospitals using employee online reviews
Source: PLoS One. 2022 Sep 14;17(9):e0274074. doi: 10.1371/journal.pone.0274074 (PMC9473622; doi:10.1371/journal.pone.0274074)
Supplement: Attachment — Submitted filename: Response to Reviewers_R1.docx [file pone.0274074.s001.docx]

Reviewer Response Sheet: PONE-D-21-07002R1

An assessment of Organisational Culture in Australian Hospitals Using Employee Online Reviews

Dear editors / reviewers,

Thank you for your comments and requests for edits and clarifications. We have responded to all comments by the reviewers and revised the manuscript, and highlighted our changes based on your inputs.

| **Reviewer /editors** | **Response** |
| --- | --- |
| Reviewer #1: The aim of this study was to analyse content of reviews posted by hospital employees on three job websites. It is understandable that performing rigorous statistics might have been a challenge given the small samples. Organizational culture is a complex concept, this study provides a baseline on which future studies could build on. Did the authors considered qualitative content analysis regarding trustworthiness? | Thank you for your comments.  Yes, we did consider qualitative content analysis. However, we have limited the qualitative thematic content analysis to deductive coding to assess whether the content of the reviews met the criteria across traditional assessment instruments and tools. So as to not infringe on copyrighted content by reproducing snippets of content from the online reviews, we did not perform inductive analysis, as we are not allowed to reproduce user reviews or extracts from these reviews within the article.  Due to the general nature of the comments, and restrictions resulting from the global pandemic, follow-up interviews with hospital staff to verify the trustworthiness of these reviews was not possible at this time. We have reflected on the limitations arising from using online reviews including the risk of bias in these reviews in lines 348-355. |
| Reviewer #2: There is a lot of details that needed to be provided to strengthen this study. A lot of statements across the study sections introduce ideas that are not well expanded or tied together. The methods section is confusing and has gaps that if revised could help improve the study, including clarity on how and why 103 reviews were extracted for the study period – sample size determination? how was data abstracted from the reviews? Providing the online review (i.e. details of the elements captured by these surveys) is also a critical addition that could be made to help provide clarity on study results and discussions. I find this study to be technically lacking with insufficient depth in the analysis and presentation of the results – the insufficient introduction also adds to this limitation. | We have updated the methods section to specify that this set of 103 reviews comprised all reviews posted by users that were intelligible, referred to the right organisation and had been moderated and posted by the specified websites. (Additions made: lines 140-142)  Data were abstracted by reading the reviews online and abstracting information as described in the methods section (p 6-10)  We cannot reproduce the surveys used on each website due to copyright restrictions. However, we have described the rating systems offered by each site and how the relevant categories were used for thematic analysis between lines 153-191.  Thank you for your inputs in helping us revise our article for improved clarity. We have added additional references and information in the introduction to elaborate on key ideas addressed in the article, between lines 81-92, 95-99 and 103-109. |
| Reviewer #3: The article is well written, methodologically sound, and relevant as it seeks to answer an important question: with the proliferation of online reviews of health care providers and health facilities, how good are these reviews at providing information related to organizational culture? The answer is that they can be quite good, with some caveats. My comments are pretty minor, and the authors should feel free to accept them or not. I thought it would be interesting to have a table that compares data/findings between former employees and current employees. I also through that in the discussion it might be worth expanding upon the risk that, with online reviews, the opinions of a "vocal" minority might end up overwhelming those of a quite majority | Thank you for your comments. We have revised our manuscript to include your suggestions.  We have included a table presenting information around the currency of employment for employees who had posted online reviews where this information was available. This has been presented in lines 273-274 and 278-280.  We have also expanded our reflection about the self-selection bias and how this could arise from a vocal minority. We have referenced relevant literature to support the additions to our manuscript (lines 348-355). |
| Editors:  In your Methods section, please provide additional information about the search performed, clarifying why only three websites were included, how they were searched, and if any eligibility criteria were applied. | We have included a section clarifying our methods (lines 132-138) with the following additional information:  These websites were chosen as they offered the option of reviewing workplaces to employees and had the most well-developed scales to collate anonymous ratings. No other job and career websites in Australia had any more reviews about the hospital network in the study at the time of conducting this study. A search was run for the name of the hospital network being studies on each of the three websites, and all reviews available that were related to any of the hospitals within this network in Australia were included in the study. |
| Editors:  Please provide additional details regarding participant consent. In the ethics statement in the Methods and online submission information, please ensure that you have specified (1) whether consent was informed and (2) what type you obtained (for instance, written or verbal, and if verbal, how it was documented and witnessed). If your study included minors, state whether you obtained consent from parents or guardians. If the need for consent was waived by the ethics committee, please include this information. | We have included a section elaborating on our ethics approval (lines 196-204). We have also elaborated on the compliance with the policies and guidelines of websites used:  Ethics approval was required for the study and was granted by the Macquarie University Medicine & Health Sciences Ethics Review Subcommittee on 21/02/2020. As all reviews analysed were anonymous, it was not possible to seek individual consent of employees who posted the online reviews. Waiver of individual consent was granted by the ethics subcommittee. Therefore, the study was limited to thematic aggregation and no content from the reviews have been reproduced in any form. Permission for analysis of the reviews using our research method was sought and granted from the online job websites where the reviews were posted. |
| Editors:  If you are reporting a retrospective study of medical records or archived samples, please ensure that you have discussed whether all data were fully anonymized before you accessed them and/or whether the IRB or ethics committee waived the requirement for informed consent. If patients provided informed written consent to have data from their medical records used in research, please include this information. | Not applicable. |
| Editors:  Your ethics statement should only appear in the Methods section of your manuscript. If your ethics statement is written in any section besides the Methods, please move it to the Methods section and delete it from any other section. Please ensure that your ethics statement is included in your manuscript, as the ethics statement entered into the online submission form will not be published alongside your manuscript. | The ethics statement has been moved to the Methods section (lines 196-204). |
| Editors:  Please ensure that your Data Availability statement reports that Terms and Conditions of the websites were complied with. | We confirm that terms and conditions of websites used in our study have been complied with and explicit permission was sought for analysis of content on the websites. Data is available on these publicly available websites. |
| Editors:  Please note that funding information should not appear in the Acknowledgments section or other areas of your manuscript. We will only publish funding information present in the Funding Statement section of the online submission form. Please remove any funding-related text from the manuscript. | Funding information has been deleted from the acknowledgements section of the manuscript. |
